# Lesion-specific impacts of lameness on milk yield and composition in Holstein dairy cows

**DOI:** 10.1016/j.vas.2026.100803

**Published:** 2026-08-06

**Authors:** Behzad Khorrami, Nadia Soltani Azar, Ali Assadi-Alamouti, Mohammad Reza Bakhtiarizadeh, Esmaeil Pourhosseini

**Affiliations:** Department of Animal and Poultry Science, Faculty of Agricultural Technology (Aburaihan), University of Tehran, Tehran, Iran

**Keywords:** Claw lesions, Dairy cows, Lameness, Milk yield, Milk composition, Somatic cell count

## Abstract

This study evaluated the overall and lesion-specific effects of clinical lameness on milk yield and milk composition traits in Holstein dairy cows under commercial dairy farm conditions. A total of 18,254 test-day records from 2049 clinically lame cows in two large commercial dairy herds located in Varamin County, Tehran Province, Iran, were included in the analysis. Milk yield, milk fat percentage, milk protein percentage, and somatic cell count (SCC) were compared between pre-lameness (within 30 days before diagnosis) and post-lameness (within 30 days after diagnosis) periods using linear mixed-effects models. Herd, parity, and days in milk (DIM) were included as fixed effects, and cow was fitted as a random effect. SCC values were log10-transformed prior to analysis.

Overall, lameness significantly reduced daily milk yield (by approximately 1.3 kg/d; *P* < 0.01). Milk fat and protein percentages showed small but statistically significant reductions following lameness onset (*P* < 0.05). Mean SCC increased from 330 × 10³ cells/mL before lameness to 369 × 10³ cells/mL after lameness, although this change was not statistically significant (*P* = 0.15). Lesion-specific analyses showed that digital dermatitis, sole ulcer, phlegmon, white line disease, and foreign-object injury were associated with significant yield reductions (*P* < 0.01), whereas hoof crack had no significant effect. Changes in milk composition varied across lesion types and were generally smaller in magnitude than yield reductions. These findings highlight the production and welfare implications of lameness and emphasize the importance of early detection and lesion-specific management strategies.

## Highlights

• Lameness was associated with measurable milk production losses in dairy cows.
• Milk fat and protein concentrations declined after lameness diagnosis.
• Somatic cell count tended to increase following lameness events.
• White line disease accounted for the largest proportion of cases.
• Early detection of lameness may mitigate short-term production losses.

## 1 Introduction

Lameness is one of the most prevalent and economically significant health disorders in dairy cattle and represents a major welfare concern in modern production systems. A recent literature review reported a mean cow-level lameness prevalence of 22.8%, with estimates ranging from 5.1% to 45.0% across studies, highlighting substantial variation associated with herd management, housing, and assessment methods (Thomsen et al., 2023). Clinical lameness adversely affects cow mobility, behavior, welfare, and productive performance and may also be associated with an increased risk of other economically important health disorders, including mastitis (Džermeikaitė et al., 2025; Hisira et al., 2025).

The negative association between lameness and milk yield has been well documented. Previous studies have shown that milk production may decline weeks before clinical diagnosis and remain depressed thereafter (Green et al., 2002; Huxley, 2013; Norring et al., 2014). Proposed mechanisms include reduced feed intake, altered lying and standing behavior, systemic inflammatory responses, and nutrient redistribution away from milk synthesis toward immune activation (Huxley, 2013; Norring et al., 2014). However, previous studies suggest that production responses to lameness may differ among lesion types because of differences in etiology, severity, and duration (Amory et al., 2008; Huxley, 2013). For example, sole ulcer and white line disease have been associated with substantial reductions in milk yield (Amory et al., 2008).

The etiology and pathophysiology of claw lesions differ substantially among lesion types, with both infectious and non-infectious disorders contributing to clinical lameness in dairy herds (Garvey, 2022). Digital dermatitis is an infectious disease caused by contagious bacteria that results in painful ulcerative lesions, whereas sole ulcer and white line disease are primarily claw horn lesions related to mechanical stress and laminitis-related processes. Phlegmon (foot rot) is an acute bacterial infection of the interdigital tissues, often accompanied by systemic inflammation (Pavlenko et al., 2011; Van Metre, 2017). In contrast, the clinical severity and production impact of hoof cracks and foreign-object injuries are more variable (Green et al., 2010; Hernandez et al., 2002). Because of these biological differences, production responses are not expected to be uniform across lesion categories.

In addition to affecting milk yield, lameness may influence milk composition. Changes in milk fat and protein percentages have been linked to changes in nutrient partitioning, metabolic stress, and feed intake (Džermeikaitė et al., 2025; Necula et al., 2024). Although SCC is primarily an indicator of mammary inflammation and is strongly influenced by intramammary infection and environmental hygiene (Zigo et al., 2020), recent evidence suggests that cows affected by claw diseases may have an increased risk of mastitis (Hisira et al., 2025). Nevertheless, relatively few field studies have simultaneously evaluated the overall and lesion-specific associations of clinical lameness with milk yield, milk composition, and SCC under commercial dairy farm conditions.

Therefore, the objective of this study was to quantify the overall and lesion-specific effects of clinically diagnosed lameness on milk yield, milk fat percentage, milk protein percentage, and SCC in Holstein dairy cows using longitudinal test-day data and mixed-effects modeling. We hypothesized that, within each lesion category, milk yield would decrease after lameness onset and would be more strongly affected than milk composition traits.

## 2 Materials and methods

### 2.1 Herds and data collection

Data were obtained from two large commercial Holstein dairy herds located in Varamin County, Tehran Province, Iran, with approximately 2140 and 2320 lactating cows, respectively. Both herds were managed under similar commercial conditions using free-stall housing systems. Cows were housed in free-stall barns with sand-bedded stalls. Manure and wet or contaminated bedding were removed twice daily, and fresh sand was added weekly to maintain adequate bedding depth. The bedding was completely replaced at six-month intervals. The alleys were cleaned every two hours using scrapers. Cows had *ad libitum* access to feed and water and were fed a total mixed ration (TMR) formulated to meet the nutrient requirements of lactating dairy cows. Test-day production records were collected between January 2021 and October 2023. After applying inclusion criteria, the final analytical dataset included 18,254 test-day records from 2049 cows diagnosed with at least one confirmed clinical lameness event during lactation.

Records within 30 days before (pre-lameness) and 30 days after (post-lameness) the date of clinical diagnosis were included. Lameness events occurring before 10 DIM were excluded to minimize confounding associated with physiological instability in early lactation. Cows were retained only if they had at least one complete test-day record within each of the pre- and post-lameness periods.

DIM values reported in descriptive statistics correspond to the retained test-day records within these ±30-day windows. Accordingly, the maximum DIM reflects the range of analytical records rather than the DIM at which lameness occurred. The availability of daily production records allowed for precise temporal alignment of observations around lameness events and minimized bias due to irregular recording intervals.

Daily monitoring was conducted by trained farm personnel, and suspected cases of lameness were evaluated by herd veterinarians. Locomotion scoring was performed using the 5-point scale described by Sprecher et al. (1997), with scores ≥3 classified as clinically lame. After claw inspection in a trimming chute, lesions were categorized into six types: digital dermatitis, sole ulcer, phlegmon, white line disease, hoof crack, and foreign-object injury (Greenough, 2007; Shearer & van Amstel, 2017). When multiple lesions were present, the primary lesion responsible for lameness was recorded.

Milk yield (kg/d), milk fat percentage, milk protein percentage, and SCC were obtained from routine herd recording systems. Daily milk yield was recorded automatically at each milking using on-farm recording systems, whereas milk composition traits were obtained from routine test-day recordings at regular intervals (every 30 ± 5 days). Milk composition analyses were performed by a certified dairy laboratory using a Milko-Scan 605 analyzer (Foss Electric, Hillerød, Denmark) based on standard infrared spectroscopy methods, and SCC was determined using a Fossomatic analyzer (FOSS Electric, Hillerød, Denmark).

Before analysis, the data were screened for completeness, biological plausibility, and consistency. Only test-day records containing complete data for all key variables (milk yield, milk fat percentage, milk protein percentage, and SCC) were included in the analyses. Cows were not excluded based on clinical or subclinical mastitis status or SCC level. However, when a cow was milked separately or its milk was withheld during treatment, one or more of these variables might not have been recorded in the routine database. In such cases, only the incomplete test-day record was excluded, whereas other complete records from the same cow remained eligible for inclusion. Implausible values, including extreme observations outside biologically reasonable ranges, were identified through descriptive screening and removed. Consistency checks were also performed to verify the alignment of test-day records with cow identification and lameness-event dates. Duplicate and inconsistent records were excluded from the final analytical dataset.

### 2.2 Statistical analysis

All analyses were performed using R software (version 4.2.0; R Foundation for Statistical Computing, Vienna, Austria). Descriptive statistics were calculated for all variables. SCC was log10-transformed prior to analysis to improve residual normality and homoscedasticity, and all statistical analyses and inferences were conducted on the transformed scale. Model assumptions were verified through residual diagnostics. For the overall analysis, test-day records were classified into pre- and post-lameness periods. Linear mixed-effects models were fitted separately for milk yield, milk fat percentage, milk protein percentage, and log10SCC:

Yijklm=μ+Periodi+Herdj+Parityk+DIMl+Cowm+εijklm

where Y represents the dependent variable, μ is the overall mean, Period, Herd, and Parity were fixed effects; DIM was included as a covariate; Cow was included as a random effect to account for repeated measurements within animal; and ε represented residual error.

A suitable variance–covariance structure was selected for each response variable based on Akaike’s Information Criterion (AIC). Model assumptions were verified using residual diagnostics.

For lesion-specific analyses, separate linear mixed-effects models were fitted within each lesion category. In these models, pre- and post-lameness measurements from the same cows were used to assess within-animal changes associated with lameness onset, while maintaining the same fixed and random effects structure described above. These analyses were intended to evaluate within-animal changes associated with each lameness type independently and were not designed for direct statistical comparisons among different lesion categories. Statistical significance was declared at *P* < 0.05.

## 3 Results

### 3.1 Descriptive statistics

Descriptive statistics of cow status, milk production and composition variables are presented in Table 1. Mean parity was 2.37 ± 1.48 and mean DIM was 168.37 ± 83.62 days. Mean milk yield was 39.89 ± 11.50 kg/d. Average milk fat and protein percentages were 3.51 ± 0.68% and 3.15 ± 0.37%, respectively. The median (Q1–Q3) SCC was 130 (74–302) × 10³ cells/mL.

**Table 1:** Descriptive statistics for cow status and production variables included in the analytical dataset.

Item	Min	Q1	Median	Mean	Q3	Max
Parity	1.00	1.00	2.00	2.37	3.00	9.00
DIM (d)	10.00	58.00	173.0	168.37	260.00	336.0
Milk yield (kg/d)	9.60	32.50	40.56	39.89	47.89	56.47
Milk fat (%)	2.24	3.02	3.44	3.51	3.91	5.65
Milk protein (%)	2.42	2.90	3.10	3.15	3.34	5.22
SCC^1^	43.00	74.00	130.00	293.33	302.00	2824.0

1 Somatic cell count (SCC) is presented on the original scale (× 10³ cells/mL) for descriptive purposes. Given its non-normal distribution, median and quartile-based statistics provide a more appropriate summary of central tendency and dispersion.

**Fig. 1:**
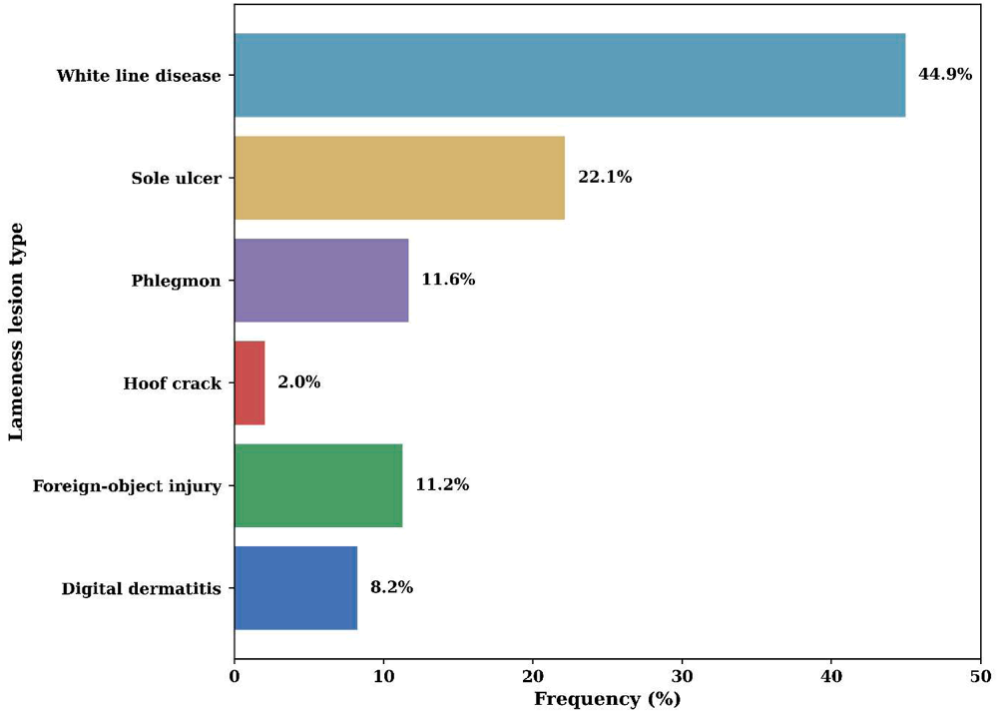
Overall frequency distribution (%) of major lameness lesion types diagnosed in Holstein dairy cows during the study period. Values are expressed as percentages of total lameness cases (n = 2049 clinically lame cows).

Fig. 1 illustrates the frequency distribution of the major lameness lesion types diagnosed in the study population. White line disease was the most prevalent lesion (n = 920 cows, 44.9%), followed by sole ulcer (n = 453 cows, 22.1%), phlegmon (n = 238 cows, 11.6%), and foreign-object injury (n = 229 cows, 11.2%). Digital dermatitis (n = 168 cows, 8.2%) and hoof crack (n = 41 cows, 2.0%) were less frequently observed and accounted for a smaller proportion of total lameness cases.

**Fig. 2:**
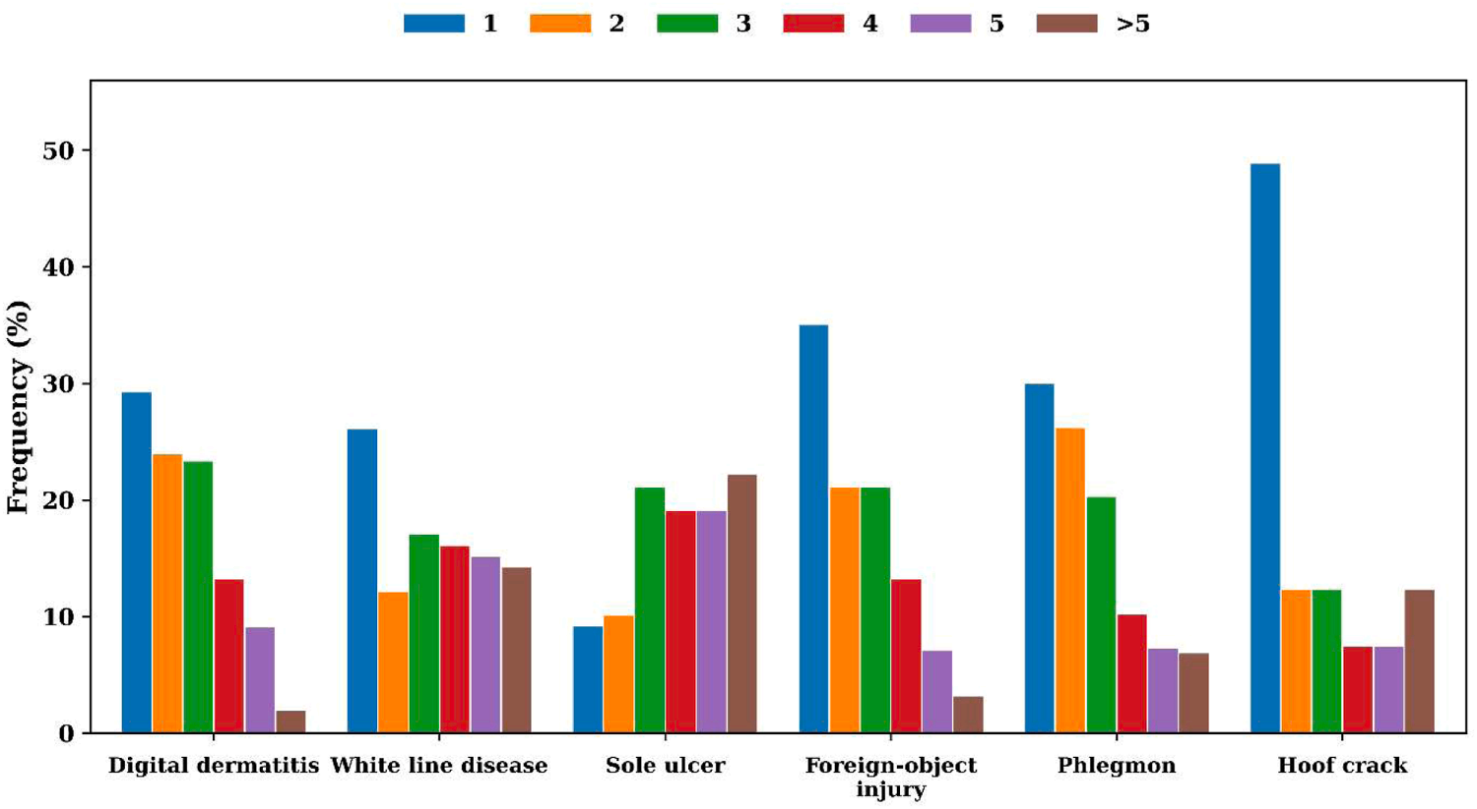
Distribution (%) of clinically lame cows across parity groups within each lameness lesion category (n = 2049). Parity groups represent the first, second, third, fourth, fifth, and > fifth lactations. Within each lesion category, bars show the proportion of cases belonging to each parity group.

Fig. 2 shows the distribution of cases across parity groups within each lameness lesion category. First-parity cows accounted for the largest proportion of digital dermatitis, hoof crack, foreign-object injury, and phlegmon cases, with the proportions generally declining in higher-parity groups. In contrast, sole ulcer cases were more concentrated in cows in their third and subsequent lactations. White line disease occurred across all parity groups, although the largest proportion of cases was observed in first-parity cows. These distributions are descriptive and do not account for differences in the number of cows at risk within each parity group.

### 3.2 Overall effect of lameness on milk production and composition

**Fig. 3:**
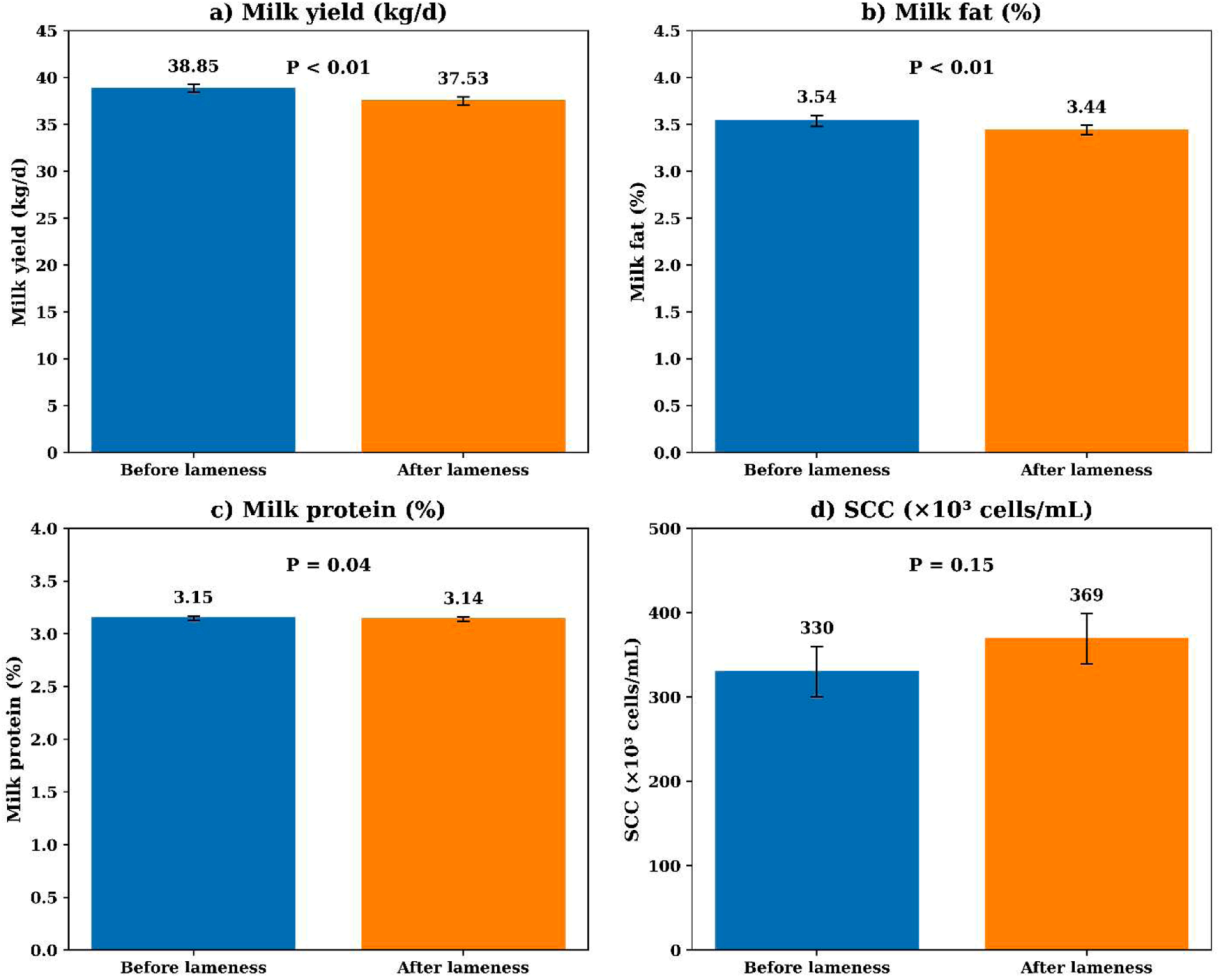
Overall changes in milk production and composition before and after the onset of clinical lameness in dairy cows: (a) milk yield (kg/d), (b) milk fat percentage, (c) milk protein percentage, and (d) somatic cell count (SCC, × 10³ cells/mL). Values in panels (a–c) are adjusted least-squares means ± SEM derived from the linear mixed-effects models. In panel (d), SCC values are presented as untransformed descriptive means ± SEM on the original scale, whereas statistical analysis was performed using log10-transformed SCC values. P-values represent within-cow comparisons between the pre- and post-lameness periods.

After adjustment for herd, parity, DIM, and repeated measurements within cow, lameness had a significant negative effect on milk production. Milk yield decreased by approximately 1.3 kg/d following lameness onset (P < 0.01; Fig. 3a). Milk fat percentage decreased by 0.10 percentage units (*P* < 0.01; Fig. 3b). Milk protein percentage also decreased by 0.01 percentage units (*P* < 0.05; Fig. 3c). Fig. 3d illustrates the changes in SCC values before and after lameness onset. Mean SCC increased from 330 × 10³ cells/mL before lameness to 369 × 10³ cells/mL after lameness; however, this change was not statistically significant after adjustment for covariates (*P* = 0.15). For clarity, SCC values are presented on the original scale in figures and tables for descriptive purposes, whereas all statistical inferences were conducted on the transformed scale.

### 3.3 Lesion-specific effects on milk production and composition

When analyzed separately by lesion category using mixed-effects models, reductions in milk yield were observed after lameness onset in several lesion types (Table 2). Milk yield declined by 6.57 kg/d in cows with digital dermatitis, 3.52 kg/d in cows with phlegmon, 2.98 kg/d in cows with sole ulcer, 1.29 kg/d in cows with white line disease, and 0.45 kg/d in cows with foreign-object injury (all *P* < 0.01). No significant reduction in milk yield was detected in cows with hoof crack.

**Table 2:** Effects of different types of lameness on milk production and composition in dairy cows^1^.

Item	Period		SEM	P-value
	Before lameness	After lameness		
**White line disease** (n = 920)				
Milk yield (kg/d)	40.02	38.73	0.531	<0.01
Milk fat, %	3.50	3.40	0.056	0.07
Milk protein, %	3.13	3.11	0.027	0.77
SCC^2^	370 (330–415)	388 (345–436)	-	0.37
**Digital dermatitis** (n = 168)				
Milk yield (kg/d)	42.11	35.54	1.447	<0.01
Milk fat, %	3.64	3.26	0.133	0.45
Milk protein, %	3.13	3.12	0.054	0.93
SCC	141 (125–159)	129 (114–146)	-	<0.01
**Foreign-object injury** (n = 229)				
Milk yield (kg/d)	37.19	36.74	1.346	<0.01
Milk fat, %	3.66	3.41	0.147	0.06
Milk protein, %	3.26	3.17	0.061	<0.01
SCC	424 (360–499)	351 (301–410)	-	0.51
**Hoof crack** (n = 41)				
Milk yield (kg/d)	39.45	39.15	1.997	0.89
Milk fat, %	3.16	3.11	0.385	0.91
Milk protein, %	3.05	3.03	0.080	<0.01
SCC	85 (72–100)	27 (22–33)	-	<0.01
**Phlegmon** (n = 238)				
Milk yield (kg/d)	41.26	37.74	1.228	<0.01
Milk fat, %	3.57	3.48	0.115	0.43
Milk protein, %	3.19	3.14	0.072	0.17
SCC	294 (268–322)	278 (253–305)	-	0.58
**Sole ulcer** (n = 453)				
Milk yield (kg/d)	42.51	39.53	0.845	<0.01
Milk fat, %	3.52	3.47	0.085	0.81
Milk protein, %	3.10	3.08	0.084	<0.01
SCC	399 (362–440)	438 (398–482)	-	0.90

1 Values for milk yield, milk fat percentage, and milk protein percentage are least-squares means derived from linear mixed-effects models including cow as a random effect to account for repeated measurements.

2 Somatic cell count (SCC) values are presented as untransformed means (× 10³ cells/mL) with 95% confidence intervals for descriptive purposes only. All statistical analyses were conducted on log10-transformed SCC values. Standard errors are not presented for SCC because statistical inference was conducted on the transformed scale.

**Fig. 4:**
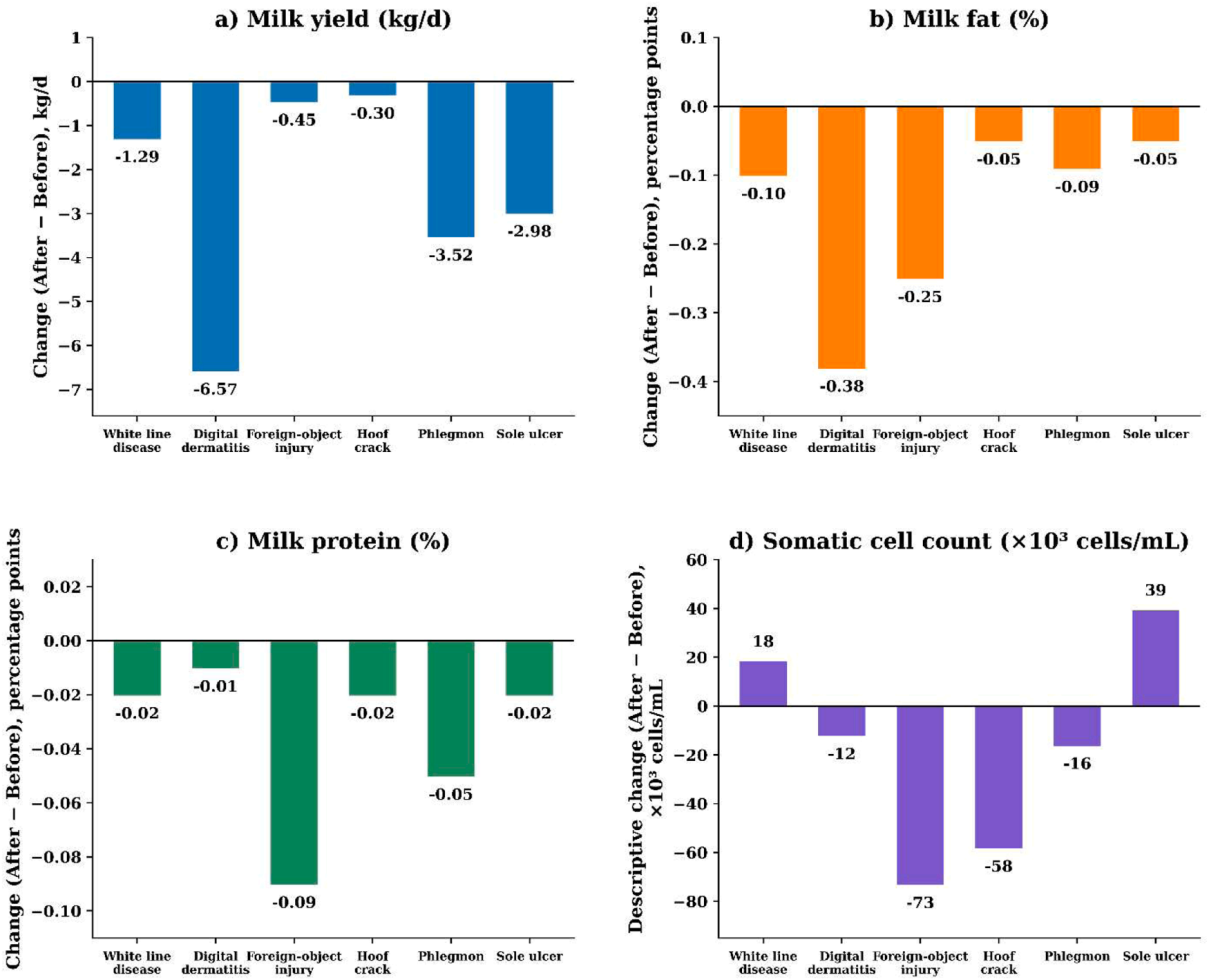
Lesion-specific changes (After − Before) in milk yield (a), milk fat percentage (b), milk protein percentage (c), and somatic cell count (SCC; d) following clinical lameness onset in Holstein dairy cows. Panels a–c show differences between model-derived least-squares means, whereas panel d shows descriptive differences in untransformed SCC means (× 10³ cells/mL); SCC analyses were performed on log10-transformed values. Positive and negative values indicate increases and decreases, respectively. Comparisons across lesion categories are descriptive; within-lesion P-values are reported in Table 2.

Milk fat percentage tended to decrease by 0.10 percentage units in cows with white line disease (*P* = 0.07) and by 0.25 percentage units in cows with foreign-object injury (*P* = 0.06). Milk protein percentage declined by 0.09, 0.02, and 0.02 percentage units in cows with foreign-object injury, hoof crack, and sole ulcer, respectively (all *P* < 0.01), whereas no significant changes were detected in cows with digital dermatitis, white line disease, or phlegmon. SCC responses varied among lesion categories. Significant decreases were observed in cows with digital dermatitis and hoof crack (*P* < 0.01), whereas no significant SCC changes were detected in cows with white line disease, sole ulcer, phlegmon, or foreign-object injury. Fig. 4 provides a visual comparison of lesion-specific changes in milk yield, milk fat percentage, milk protein percentage, and SCC before and after lameness.

## 4 Discussion

The present study evaluated the overall and lesion-specific effects of clinical lameness on milk yield and milk composition in Holstein dairy cows managed in commercial dairy herds. The findings demonstrate that lameness is associated with a measurable reduction in milk yield and modest alterations in milk composition, with production responses differing across lesion categories. These findings are consistent with previous reports indicating that lame cows often experience decreased productivity due to compromised welfare and metabolic stress (Green et al., 2002; Hernandez et al., 2002; Warnick et al., 2001).

Although the study population consisted predominantly of mid-lactation cows, the reduction in milk yield remained evident after adjustment for DIM, suggesting that the observed decline was associated with lameness itself rather than normal lactational progression. The relatively high average milk yield observed in the study likely reflects the overall production level of the commercial herds included in the analysis, similar to observations reported in intensively managed dairy systems (Bicalho et al., 2008; Cook et al., 2016).

The observed reduction in milk yield following lameness diagnosis is consistent with the hypothesis that locomotion disorders negatively affect productive performance through both behavioral and physiological mechanisms. Lame cows typically exhibit reduced feed intake, altered lying and standing behavior (Green et al., 2002; Huxley, 2013; Norring et al., 2014), and increased energy expenditure associated with pain and stress (Norring et al., 2014; Van Metre, 2017). These changes may contribute to negative energy balance and a redistribution of nutrients away from milk synthesis toward maintenance and immune function (Necula et al., 2024). The magnitude of yield reduction observed in the present study is comparable to previous reports (Relun et al., 2013; Warnick et al., 2001). Differences among studies are likely related to variation in herd management, lesion severity, environmental conditions, and stage of lactation (Bicalho et al., 2008; Huxley, 2013; Necula et al., 2024).

In contrast to milk yield, changes in milk fat and protein percentages were relatively small, suggesting that lameness primarily affects overall milk volume rather than substantially altering milk synthesis pathways. The slight reductions observed may reflect alterations in rumen fermentation and nutrient partitioning associated with reduced feed intake and metabolic stress (Džermeikaitė et al., 2025; Necula et al., 2024). However, the biological significance of these compositional changes appears limited compared with the reduction observed in milk yield. Similar findings have been reported previously, where milk yield was more sensitive to lameness and health disorders than milk composition traits (Džermeikaitė et al., 2025; Green et al., 2010).

Although SCC increased numerically after lameness onset, this change was not statistically significant, suggesting that the relationship between lameness and udder health is complex and likely indirect (Huxley, 2013). Painful claw lesions may induce physiological stress and systemic inflammation, including increased cortisol concentrations and pro-inflammatory mediators, which may alter immune competence and increase susceptibility to intramammary infection (Necula et al., 2024). In addition, prolonged lying time and impaired mobility may increase teat contact with contaminated bedding and environmental mastitis pathogens, particularly under suboptimal hygiene conditions (Hisira et al., 2025; Zigo et al., 2020). Consistent with these mechanisms, Hisira et al. (2025) reported a greater occurrence of mastitis in cows affected by claw diseases. Nevertheless, SCC is primarily determined by intramammary infection, and shared factors such as housing hygiene, mastitis prevalence, pathogen exposure, and herd management may influence the observed association. Therefore, the non-significant increase observed here does not establish a direct causal relationship and should be interpreted cautiously, consistent with the variable associations previously reported between lameness and SCC (Archer et al., 2011; Huxley, 2013).

The lesion-specific analyses provide further insight into the heterogeneity of lameness-associated production responses. In the present study, notable reductions in milk yield were observed in cows affected by digital dermatitis, sole ulcer, white line disease, and phlegmon. These findings are biologically plausible because digital dermatitis causes painful ulcerative lesions (Garvey, 2022), sole ulcer and white line disease involve disruption of the claw horn and damage to the underlying corium (Shearer & van Amstel, 2017), and phlegmon is characterized by acute infection and inflammation of the interdigital tissues (Van Metre, 2017). The resulting pain, inflammation, and impaired locomotion may alter feeding behavior and reduce milk production.

The observed milk yield reductions were generally consistent with previous reports on claw horn lesions and digital dermatitis (Amory et al., 2008; Pavlenko et al., 2011; Relun et al., 2013). Reductions associated with sole ulcer (2.98 kg/d) and white line disease (1.29 kg/d) were comparable to those reported by Amory et al. (2008), whereas the reduction for digital dermatitis (6.57 kg/d) exceeded most previous estimates. Hernandez et al. (2002) also reported lower milk production in cows affected by interdigital phlegmon than in healthy cows. Differences among studies may reflect variation in lesion type and severity, disease stage, herd management, and analytical approach (Džermeikaitė et al., 2025; Necula et al., 2024), emphasizing the importance of lesion-specific evaluation and management.

Significant reductions in milk yield were also detected in cows with white line disease and foreign-object injury. The variability observed in some lesion categories, particularly foreign-object injury, may have contributed to larger standard errors and reduced sensitivity for detecting statistically significant differences. No detectable reduction in milk yield was observed in cows with hoof crack; however, this finding should be interpreted cautiously because this lesion category included only 41 cows, which may have limited statistical power and increased the influence of individual-animal variability. These findings emphasize the importance of considering lesion type when evaluating the economic and welfare consequences of lameness in dairy herds.

Assuming that the short-term economic consequences of lameness are largely driven by reduced milk production, the lesion-specific reductions observed in the present study indicate considerable variation in daily milk-revenue loss. If P represents the farm-gate price per kilogram of milk in the relevant market, the estimated daily milk-revenue loss per affected cow would be 6.57P for digital dermatitis, 3.52P for phlegmon, 2.98P for sole ulcer, 1.29P for white line disease, and 0.45P for foreign-object injury. Although the estimated reduction associated with hoof crack would correspond to 0.30P, its effect on milk yield was not statistically significant and should not be interpreted as a confirmed economic loss. This price-adjustable approach avoids reliance on location- and time-specific milk prices and allows the estimates to be applied across different production systems. These estimates represent losses from reduced milk sales only and do not include treatment, labor, reproductive, or culling costs; therefore, the total economic burden of lameness may be greater.

Several limitations of the study should be acknowledged. The absence of a contemporaneous non-lame control group limits causal interpretation. However, the within-cow comparison design reduced between-animal variability and improved the estimation of temporal changes associated with lameness. In addition, lesion severity was not quantified, which may have contributed to variability in production responses among lesion categories. Although DIM was included as a covariate in the statistical models, the effects of lameness were not evaluated separately across lactation stages and warrant further investigation. Moreover, because milk from cows with clinical mastitis may have been withheld or recorded separately during treatment, some test-day records corresponding to acute mastitis episodes may not have been available in the routine herd database. Consequently, acute mastitis periods may be underrepresented in the analytical dataset, which should be considered when interpreting the SCC and milk-composition findings. Despite these limitations, the large dataset, longitudinal structure, and use of mixed-effects models strengthen the robustness and practical relevance of the findings.

Overall, the production and welfare consequences of lameness varied among lesion categories, emphasizing the importance of lesion-specific evaluation. Timely detection and treatment may reduce milk-production losses, improve cow welfare, and support effective herd management.

## 5 Conclusion

This large-scale study showed that clinical lameness was associated with reduced milk yield and modest changes in milk composition, with digital dermatitis, phlegmon, and sole ulcer representing the highest-priority lesions because of their greater production losses. Preventive strategies should include appropriate foot-bathing, hygiene, and biosecurity for digital dermatitis; clean, dry, and trauma-free walking surfaces for phlegmon and foreign-object injuries; and regular functional hoof trimming, comfortable stalls, well-maintained flooring, and appropriate nutritional management for sole ulcer, white line disease, and hoof cracks. Routine locomotion scoring, hoof inspection, and prompt lesion-specific treatment should be integrated into herd health programs to reduce production losses and improve cow welfare.

## Ethical statement

The study protocol was reviewed and approved by the Animal Research Committee at the University of Tehran. All procedures complied with the journal’s ethical policies and EU standards for the protection of animals used for scientific purposes.

## Ethical policies

The authors confirm that the ethical policies of the journal, as noted on the journal's author guidelines page, have been adhered to and the appropriate ethical review committee approval has been received. The authors confirm that they have followed EU standards for the protection of animals used for scientific purposes.

## Ethics declaration

This study was conducted in accordance with the following guidelines for animal welfare and/or reporting: This observational study used routinely collected herd records and clinical data from commercial dairy farms. No experimental interventions were performed, and the study complied with applicable animal-welfare standards. This study was approved by the Animal Research Committee at the University of Tehran.

## Funding statement

This research did not receive any specific grant from funding agencies in the public, commercial, or not-for-profit sectors.

## CRediT authorship contribution statement

**Behzad Khorrami:** Writing – review & editing, Writing – original draft, Supervision, Project administration, Investigation, Conceptualization. **Nadia Soltani Azar:** Project administration, Methodology, Investigation, Data curation. **Ali Assadi-Alamouti:** Supervision, Project administration, Methodology, Conceptualization. **Mohammad Reza Bakhtiarizadeh:** Validation, Formal analysis, Data curation. **Esmaeil Pourhosseini:** Software, Formal analysis, Data curation.

## Declaration of competing interest

The authors declare that they have no known competing financial interests or personal relationships that could have appeared to influence the work reported in this paper.
